# Efficient recovery of proteins from multiple source samples after trizol^^®^^ or trizol^^®^^LS RNA extraction and long-term storage

**DOI:** 10.1186/1471-2164-14-181

**Published:** 2013-03-15

**Authors:** André ES Simões, Diane M Pereira, Joana D Amaral, Ana F Nunes, Sofia E Gomes, Pedro M Rodrigues, Adrian C Lo, Rudi D'Hooge, Clifford J Steer, Stephen N Thibodeau, Pedro M Borralho, Cecília MP Rodrigues

**Affiliations:** 1 Research Institute for Medicines and Pharmaceutical Sciences (iMed.UL), Faculty of Pharmacy, University of Lisbon, Lisbon, Portugal; 2 Laboratory of Biological Psychology, University of Leuven, Leuven, Belgium; 3 Department of Medicine, and Department of Genetics, Cell Biology and Development, University of Minnesota, Minneapolis, MN, USA; 4 Department of Laboratory Medicine and Pathology, Mayo Clinic, Rochester, MN, USA; 5 Department of Biochemistry and Human Biology, Faculty of Pharmacy, University of Lisbon, Lisbon, Portugal

**Keywords:** Protein extraction, Sonication, TRIzol^®^ protein isolation, Long-term sample storage in TRIzol^®^, Ponceau S as loading control in immunobloting

## Abstract

**Background:**

Simultaneous isolation of nucleic acids and proteins from a single biological sample facilitates meaningful data interpretation and reduces time, cost and sampling errors. This is particularly relevant for rare human and animal specimens, often scarce, and/or irreplaceable. TRIzol^®^ and TRIzol^®^LS are suitable for simultaneous isolation of RNA, DNA and proteins from the same biological sample. These reagents are widely used for RNA and/or DNA isolation, while reports on their use for protein extraction are limited, attributable to technical difficulties in protein solubilisation.

**Results:**

TRIzol^®^LS was used for RNA isolation from 284 human colon cancer samples, including normal colon mucosa, tubulovillous adenomas, and colon carcinomas with proficient and deficient mismatch repair system. TRIzol^®^ was used for RNA isolation from human colon cancer cells, from brains of transgenic Alzheimer’s disease mice model, and from cultured mouse cortical neurons. Following RNA extraction, the TRIzol^®^-chloroform fractions from human colon cancer samples and from mouse hippocampus and frontal cortex were stored for 2 years and 3 months, respectively, at −80°C until used for protein isolation.

Simple modifications to the TRIzol^®^ manufacturer’s protocol, including Urea:SDS solubilization and sonication, allowed improved protein recovery yield compared to the TRIzol^®^ manufacturer’s protocol. Following SDS-PAGE and Ponceau and Coomassie staining, recovered proteins displayed wide molecular weight range and staining pattern comparable to those obtainable with commonly used protein extraction protocols. We also show that nuclear and cytosolic proteins can be easily extracted and detected by immunoblotting, and that posttranslational modifications, such as protein phosphorylation, are detectable in proteins recovered from TRIzol^®^-chloroform fractions stored for up to 2 years at −80°C.

**Conclusions:**

We provide a novel approach to improve protein recovery from samples processed for nucleic acid extraction with TRIzol^®^ and TRIzol^®^LS compared to the manufacturer`s protocol, allowing downstream immunoblotting and evaluation of steady-state relative protein expression levels. The method was validated in large sets of samples from multiple sources, including human colon cancer and brains of transgenic Alzheimer’s disease mice model, stored in TRIzol^®^-chloroform for up to two years. Collectively, we provide a faster and cheaper alternative to the TRIzol^®^ manufacturer`s protein extraction protocol, illustrating the high relevance, and wide applicability, of the present protein isolation method for the immunoblot evaluation of steady-state relative protein expression levels in samples from multiple sources, and following prolonged storage.

## Background

The understanding of the molecular events taking place in cells and tissues under physiological or pathological conditions is the major goal of molecular biology. With this purpose, genome, transcriptome and proteome analysis are common procedures, made possible through DNA, RNA and protein extraction and analysis. Currently, multiple protocols and reagents can be used for DNA, RNA or protein extraction and isolation. However, the development of reagents that allow the routine isolation of DNA, RNA and proteins from a single sample, such as TRIzol^®^, has contributed to maximizing the information that can be extracted from valuable research samples.

TRIzol^®^ and TRIzol^®^LS are ready-to-use commercially available reagents, consisting of a monophasic solution of phenol and guanidine isothiocyanate, allowing the isolation of DNA, RNA and proteins from cell culture and tissue samples of multiple sources [1]. Numerous publications report the use of TRIzol^®^ for nucleic acid isolation. In contrast, only few studies report the utility of this reagent for protein isolation, mainly due to technical difficulties in the solubilization of the isolated protein fraction [2-4]. One of the major limitations in research using human clinical and animal model samples is the amount of material available, which is most often scarce, allowing limited downstream applications. Therefore, the collection of a proper amount of nucleic acids and proteins for subsequent analysis becomes crucial. In addition, the simultaneous isolation of DNA, RNA and proteins from a single sample facilitates the meaningful interpretation and correlation of genome, transcriptome and proteome data, while reducing time, cost and sampling error [3-5]. An additional advantage reported using TRIzol^®^ for protein isolation is related to the reduced loss of protein, compared with previous sample preparation methods, by preventing protein aggregation during salt clearance [6].

In the present study, we report the use of the TRIzol^®^LS reagent for protein isolation from 284 human colon cancer (CC) samples stored at −80°C for 2 years in TRIzol^®^-chloroform, following RNA extraction. We also report the use of TRIzol^®^ reagent for protein isolation from amyloid precursor protein (APP)/Presenilin 1 (PS1) double-transgenic (APP/PS1) and wild-type mice hippocampus and cortex brain regions, as well as from mouse primary cortical neurons. Previous studies have reported the extraction of proteins from samples stored in phenol-ethanol supernatants for up to 6 months at −80°C [3], and up to 3 years at −20°C [2], by implementing modifications to the TRIzol^®^ manufacturer’s protocol, to overcome difficulties in protein solubilisation. Interestingly, sample lysis by automated frozen disruption (AFD) prior to RNA and protein extraction using TRIzol^®^ was shown to provide comparable RNA, and higher protein recovery yield, as compared to direct sample homogeneization in TRIzol^®^, and may be preferred in the case of coupled RNA and protein analysis [4]. However, TRIzol^®^ is widely used for total RNA extraction, with sample disruption being mostly performed directly in TRIzol^®^, with or without mechanical sample disruption. Furthermore, in most cases, the remainder fractions containing DNA and protein are very often discarded, thus limiting information obtainable from these samples. In this study, we present an alternative and simple modification to the TRIzol^®^ and TRIzol^®^LS manufacturer’s protocol, applicable to samples processed directly in TRIzol^®^ for nucleic acid extraction. This resulted in a fast solubilisation of protein pellets. 1D SDS-PAGE gel separation provided protein patterns comparable to those obtained with other commonly used methods for total protein extraction, following Ponceau and Coomassie staining, although with lower protein recovery. Importantly, our modified protocol displayed superior recovery performance and yield, compared to the TRIzol^®^ manufacturer’s protocol, and allowed the subsequent immunoblot detection and relative evaluation of the steady-state levels of relevant proteins in human cancer, and Alzheimer's disease (AD), including post-translationally modified (phosphorylated), as well as cytosolic and nuclear proteins, although we cannot attest the method would be suitable for quantitative protein analysis. This highlights the relevance of the present method and its utility for research efforts involving small amounts of sample material, from human cancer samples to mouse models of disease, and its applicability to the immunoblot evaluation of steady-state relative protein expression levels in samples from multiple sources, following prolonged storage.

## Methods

### Biological samples

#### Human colon cancer tissue

Specimens of human colon were obtained from a previous study, where total RNA was extracted to evaluate microRNA expression profiles [7]. Briefly, specimens from patients with CC or polyps were snap frozen in liquid nitrogen at the time of collection and then stored at −80°C for later use. Normal areas of colonic epithelium were obtained from either the margin of resection or adjacent to the tumor. Rectal cancers were excluded. Polyps were evaluated for histologic type, with only tubulovillous adenomas selected for study. Pathologic tumor staging was classified according to Dukes’ criteria [8]. DNA mismatch repair (MMR) status was evaluated as previously described [7]. Further, all samples included in the present study were anonymized, and comprised 284 colon samples, including 53 normal colon, 39 adenomas, 51 dMMR carcinomas, and 141 pMMR carcinomas.

The Mayo Clinic Institutional Review Board reviewed and approved for human studies the protocol entitled “The Identification and Validation of miRNA Signature Profiles as Biomarkers for Colon Cancer Progression” from Dr. Stephen N. Thibodeau. The Committee noted that the human studies aspects involve the use of samples collected under IRB-approved protocols. The Committee determined that the consenting process allows for future use of the samples as exemplified in the current protocol. The majority of patients provided written informed consent. For those who did not, samples were anonymized.

#### Mouse brain tissue

Specimens of mouse brain were also made available from a previous study, where total RNA was extracted to evaluate microRNA expression profiles [9]. Eight-month old male APP/PS1 double-transgenic mice [10] and their wild-type littermates were used in this study, comprising 5 cortex and 5 hippocampus samples from WT and APP/PS1 animals. Animals were sacrificed by cervical dislocation and the brains removed. All protocols have been reviewed and approved by the animal ethics committee of the University of Leuven. One hemisphere was dissected into hippocampus and frontal cortex, and snap-frozen for subsequent RNA and protein extraction.

#### Human colon cancer cells

HCT116 human colorectal carcinoma cells were grown in Dulbecco’s modified Eagle’s medium (DMEM) (Invitrogen Corp.) supplemented with 10% fetal bovine serum and 1% antibiotic/antimycotic solution (Invitrogen Corp.) and maintained at 37°C in a humidified atmosphere of 5% CO_2_, as previously described [11,12].

### Sample processing and protein extraction

#### Human colon cancer tissue

Frozen tissue sizes equivalent to 7 mm^2^ and 10-μm thick were sectioned and placed in a vial containing 400 μl of RLT buffer (QIAGEN, Chatsworth, CA) including 4 μL of β-mercaptoethanol. The vials were stored at −80°C until utilized for RNA extraction using TRIzol^®^LS (Invitrogen Corp.) according to the manufacturer’s instructions [7]. Following RNA extraction, after the addition of chloroform and phase separation with removal of the aqueous phase containing the RNA, the interphase and the organic phase of the TRIzol^®^-chloroforms were stored at −80°C for 2 years, until processed for protein extraction.

#### Mouse brain tissue

Frozen hippocampus and frontal cortex tissues were stored at −80°C until utilized for RNA extraction using TRIzol^®^ (Invitrogen Corp.) according to the manufacturer’s instructions. Following RNA extraction for use in a study to evaluate AD-related gene expression [9], the interphase and the organic phase of the TRIzol^®^-chloroform extracts were stored at −80°C for 3 months, until processed for protein extraction.

### Total protein isolation

To compare protein extraction methods, HCT116 cells were plated in 100 mm plates at 10^6^ cells per plate. Forty-eight hours later, each plate was individually scraped, and cells collected together with cell culture supernatants, to standardize inputs for protein extraction. Next, cell suspensions were centrifuged at 500 g, 5 min at 4°C, and the supernatants were discarded. Cell pellets were resuspended in the respective protein extraction reagent for each method, including Cell Disruption Buffer using the commercial mirVana™ PARIS™ kit (#AM1556; Invitrogen Corp.); A+2X Buffer using a standardized laboratory protocol [11-13]; or TRIzol^®^ using the TRIzol^®^ manufacturer’s protocol with and without modifications.

#### mirVana™ PARIS™

Ice-cold cell disruption buffer was added to cell pellets, followed by vigorous vortexing to improve cell lysis. Samples were then incubated on ice for 10 min. Subsequently, samples were sonicated, and cleared by centrifugation at 3,200 g or 10,000 g for 10 min, at 4°C. The clear supernatants containing the total protein extracts were transferred to a fresh tube and stored at -80°C.

#### A+2X buffer

Ice cold 1:1 solution of buffer A (10 mM Tris–HCl, pH 7.6, 5 mM MgCl2, 1.5 mM KAc, 2 mM dithiothreitol (DTT) and Halt™ Protease and Phosphatase inhibitor cocktail, EDTA-free (#78445, Thermo Scientific)) and buffer 2X (10 mM Tris–HCl pH 7.6, 1% Nonidet-P40 and Halt™ Protease and Phosphatase inhibitor cocktail) was added to cell pellets, and the samples were homogeneized by vigorous vortexing and incubated on ice for 30 min. Next, samples were sonicated, centrifuged at 3,200 g or 10,000 g for 10 min, at 4°C. The clear supernatants containing the total protein extracts were transferred to a fresh tube and stored at −80°C.

#### TRIzol^®^

Total protein extracts were obtained following the manufacturer’s instructions, until the protein solubilisation step. In parallel, protein pellets were solubilized in: 1% SDS, with and without 1 h incubation in a 50°C water bath; 1:1 solution of 1% SDS and 8 M urea, with and without 1 h of sample incubation in a 50°C water bath; and 1:1 solution of 1% SDS and 8 M urea, with sonication (modified TRIzol protocol). Samples were clarified by centrifuging at 3,200 g or 10,000 g to sediment insoluble material. The clear supernatants containing the total protein extracts were transferred to fresh tubes and stored at −80°C.

#### Modified TRIzol protocol

**Figure 1:**
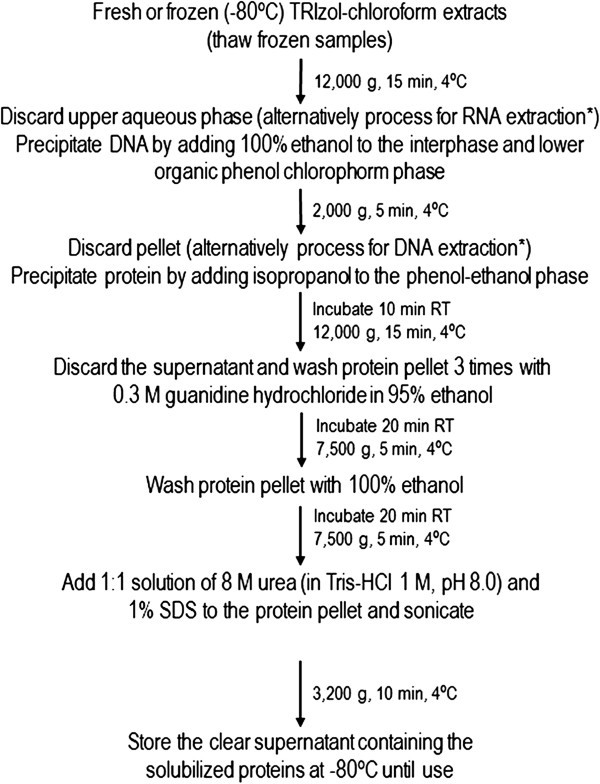
**Modified TRIzol protein extraction.** The protocol followed for protein extraction from the TRIzol^®^-chloroform fractions is depicted as a flow chart. The extracted proteins were stored at −80°C until further analysis. *According to TRIzol^®^ manufacturer’s instructions.

The flow chart in Figure 1 depicts the protocol followed. This protein extraction protocol was common to all samples from human colon and colon cancer cells, mouse brain, and mouse primary cortical neurons, adjusting the volumes of reagents to TRIzol^®^LS or TRIzol^®^ processed samples, according to the manufacturer’s instructions. Briefly, the tube containing the organic phase and a small portion of aqueous phase (TRIzol^®^-chloroform fractions) from previous phase separation was centrifuged at 12,000 g for 15 min at 4°C, and the remaining aqueous phase supernatant was removed and discarded. Subsequently, 100% ethanol was added to precipitate DNA. Tubes were mixed by inversion and centrifuged at 2,000 g for 5 min, at 4°C. The phenol-ethanol supernatant was removed to 2 mL tubes, for protein extraction. Protein was precipitated with isopropanol, followed by mixing, and 10 min incubation at room temperature. Subsequently, samples were centrifuged at 12,000 g for 10 min and the supernatant discarded. Protein pellets were next washed three times with 0.3 M guanidine hydrochloride in 95% ethanol. In each wash, tubes were vigorously shaken, incubated at room temperature for 20 min followed by centrifugation at 7,500 g for 5 min at 4°C. After the final wash and spin, 100% ethanol was added, and samples incubated at room temperature for 20 min, followed by a final centrifugation at 7,500 g for 5 min at 4°C. The supernatant was removed and a 1:1 solution of 1% SDS and 8 M urea in Tris–HCl 1 M, pH 8.0 was added to the protein pellets, followed by 5 cycles of 15 sec sonication and 30 sec ice incubation, to solubilize the protein, a modification from the 1% SDS resuspension solution recommended in the manufacturer’s protocol. Sonication was performed using a compact ultrasonic device with amplitude adjusted to 80% and pulse to 90% (model UP100H, Hielscher Ultrasonics GmbH, Teltow, Germany - 100 watts, ultrasonic frequency 30 kHz). Next, the samples were centrifuged at 3,200 g for 10 min at 4°C, to sediment insoluble material. The supernatant containing the solubilized proteins was transferred to a fresh tube and stored at −80°C.

### Protein quantification

Protein concentration was determined using the Bradford method with the Bio-Rad Protein Assay kit (Bio-Rad, California, USA), according to the manufacturer’s instructions. This method involves the addition of an acidic dye, Coomassie^®^ Brilliant Blue G-250, and subsequent absorbance measurement at 595 nm. Bovine albumin was used as standard (New England Biolabs Inc., New England). Absorbance was measured in a UNICAM UV/Vis 2 spectrophotometer (Speck & Burke Analytical, Clackmannanshire, Scotland) at 595 nm. The protein concentrations were determined from interpolation of absorbance on a linear regression standard curve.

### Immunobloting

#### Human colon cancer tissue and human colon cancer cells

Steady-state protein expression levels were determined by immunoblot analysis. Briefly, 40 μg of total protein extracts were separated on 8% sodium dodecyl sulphate-polyacrylamide gel electrophoresis (SDS-PAGE). After electrophoretic transfer onto nitrocellulose membranes, immunoblots were blocked with 5% milk solution for 30 min. Subsequently, blots were incubated overnight at 4°C with primary rabbit anti p-Akt, Akt, NF-κB (p65), IκB-α, and PARP (#sc-7985-R, #sc-8312, #sc-372, #sc-371, #sc-7150, respectively) and with primary mouse anti GAPDH, p53 (#sc-32233 and #sc-126, respectively ), all from Santa Cruz Biotechnology, Inc. (Santa Cruz, CA, USA), and β-actin (#A-5441; Sigma-Aldrich). Next, immunoblots were incubated with anti-rabbit or anti-mouse secondary antibody conjugated with horseradish peroxidase (Bio-Rad Laboratories) for 3 h at room temperature. Finally, blots were processed for protein detection using Super Signal™ substrate (Pierce, Rockford, IL, USA). Ponceau S dye at 0.2% (w/v) (Merck KGaA, Darmstadt, Germany) staining of immunoblots was used as a loading control.

#### Mouse brain tissue

Sixty μg of total protein extracts from dissected hippocampus and frontal cortex were separated on 6 and 12% SDS-PAGE. Electrophoretic transfer and immunoreacted protein bands were visualized as described above for human colon samples. The primary antibodies included rabbit polyclonal antibodies reactive to ApoE and SORLA (#sc-98574 and #sc-33822, respectively; Santa Cruz Biotechnology), and mouse monoclonal antibody reactive to β-Actin (Sigma-Aldrich). β-Actin or Ponceau S dye staining was used as loading controls. In addition, 60 μg of total protein extracts from hippocampus were electrophoretically resolved on 10-20% Tris-Tricine gels (BioRad Laboratories), and human APP, APP-C-terminal fragment β (APP-CTF-β) and amyloid-β (Aβ) peptide were identified using a primary mouse monoclonal antibody reactive to human Aβ (6E10; Signet, Emeryville, CA). Ponceau S dye staining was used as loading control.

### Sandwich ELISA

Total protein extracts from dissected hippocampus and frontal cortex from APP/PS1 mice were also used to determine Aβ_1–42_ to Aβ_1–40_ ratios. Human total Aβ_1–40_ or Aβ_1–42_ content was measured by sandwich ELISA (#EZBRAIN40 and #EZBRAIN42, respectively; Millipore Corporation, Billerica, MA, USA) according to the manufacturer’s instructions, with minor modifications in the sample preparation step. Briefly, 10 ng of total protein extracts were adjusted to a final volume of 50 μL with the reagent Standard and Sample Diluent, supplied by the kit. All subsequent assay procedure was carried out following the manufacturer’s instructions.

### Densitometry and statistical analysis

The relative intensities of protein bands were analyzed using the densitometric analysis program Quantity One version 4.6 (Bio-Rad Laboratories). All data are expressed as the mean ± SEM of similar samples, from three independent experiments. Protein band intensities for each human colon cancer sample, in all blots, were normalized to the mean protein band intensities from duplicate HCT116 total protein sample present in the same blot, and similarly included in all blots. HCT116 protein extracts used as internal normalization controls in each blot were all from the same batch. Statistical significance was evaluated using the non-parametric statistical analysis Kruskal-Wallis test with Dunn’s post-test for selected comparisons. Values of p < 0.05 were considered statistically significant.

## Results and discussion

### Performance of modified TRIzol protocol

**Figure 2:**
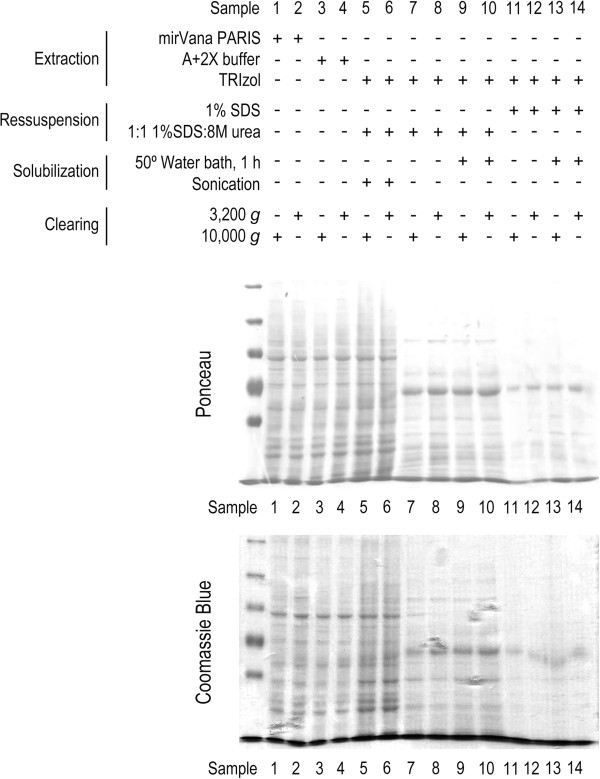
**Performance of the modified TRIzol protocol evaluated by Ponceau S and Coomassie staining.** To compare the performance of the modified TRIzol protocol versus the TRIzol^®^ manufacturer’s, mirVana^TM^ PARIS^TM^ kit and standardized laboratory (A+2X Buffers) protocols, 10^6^ HCT116 cells were plated in 14 independent 100 mm plates (one per experimental setting: lanes 1 to 14), and cells were processed for total protein extraction 48 h after plating. Next, 40 μg of total protein extracts were separated on 8% SDS-PAGE and transferred onto nitrocellulose membrane. Ponceau S staining of the nitrocellulose membrane following electrophoretic transfer of proteins, and Coomassie blue staining of total protein extracts following 8% SDS-PAGE gel separation.

**Figure 3:**
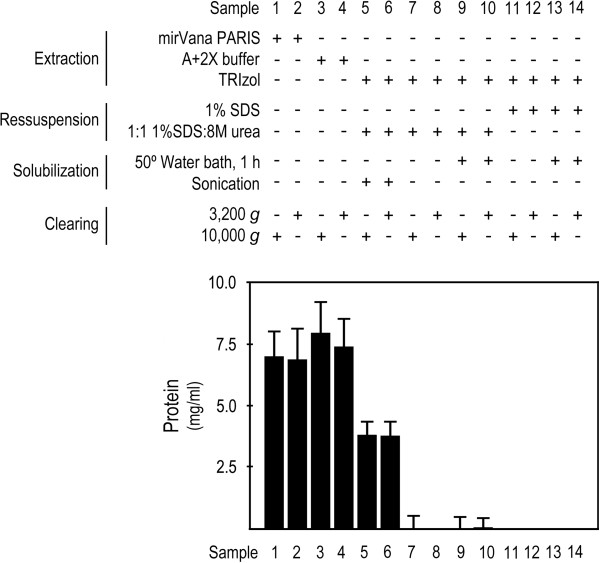
**Performance of the modified TRIzol protocol assessed by total protein extract protein recovery (mg mL**^**-1**^**).** To compare the performance of the modified TRIzol protocol versus the TRIzol^®^ manufacturer’s, mirVana^TM^ PARIS^TM^ kit and standardized laboratory (A+2X Buffers) protocols, 10^6^ HCT116 cells were plated in 14 independent 100 mm plates (one per experimental setting: lanes 1 to 14), and cells were processed for total protein extraction 48 h after plating. Protein extracts were processed in 100 μl of extraction buffer (samples 1–4), or resuspended in 100 μl of buffer (samples 5–14). Modified TRIzol protocol allowed up to ~50% recovery of total protein content in comparison with mirVana^TM^ PARIS^TM^ kit and standardized laboratory (A+2X Buffers), but importantly allowed a much higher protein recovery compared to the TRIzol manufacturer`s protocol, whose recovery yield was not suitable for downstream applications.

In the present study, we tested two simple modifications to the TRIzol^®^ and TRIzol^®^LS manufacturer’s protocol, which resulted in a fast, and almost complete, solubilization of the protein pellet. The modified protocol involves the use of a 1:1 mixture of 1% SDS and 8 M urea as protein solubilization solution, and requires sonication. Different protein extraction protocols make use of different centrifugation speeds to clarify protein samples by sedimenting insoluble materials, in the final step of the extraction protocol. We showed that no significant differences in protein patterns were detected between protein samples recovered following 3,200 g or 10,000 g centrifugation, as revealed by Ponceau and Coomassie stainings (Figure 2). To evaluate protein recovery, we compared the total amount of protein obtained with modified TRIzol protocol, with that obtained using commercial (mirVana™ PARIS™), standardized laboratory (A+2X buffers), and TRIzol^®^ total protein extraction methods. The results highlight the relevance of the sonication step, where the amount of protein recovered from the modified TRIzol protocol is much higher compared to the same protocol without sample sonication (Figure 3, lanes 5 and 6, versus lanes 7 to 10), and to the TRIzol^®^ and TRIzol^®^LS manufacturer’s protocols, which resulted in undetectable, or very low protein recovery, even after 1 h incubation at 50°C (Figure 3, lanes 5 and 6, versus lanes 11 to 14). However, in comparison with the commercial mirVana^TM^ PARIS^TM^ and standardized extraction protocols, our protocol allows the recovery of ~ 50% of total protein collected by these methods (Figure 3, lanes 5 and 6, versus lanes 1 to 4). Collectively, these results show that our modification to the Trizol^®^ manufacturer’s protocol greatly improved protein recovery from TRIzol^®^ processed samples, where the sample sonication step seemed to play an important role.

In a transcriptomic and proteomic analysis performed using small samples of rat spinal cord, it was shown that although providing similar RNA recovery yield, the protein recovery yield can be increased by processing samples using AFD followed by protein extraction using TRIzol^®^, as compared to direct sample homogenization in TRIzol^®^[4]. Further, protein recovery yield using AFD followed by extraction of proteins with TRIzol^®^ was ~ 5% lower than protein extraction using AFD followed by CHAPS/urea protein extraction [4]. However, direct sample homogeneization in TRIzol^®^ also provided a much lower protein recovery yield compared to both protein extractions using AFD, being ~ 20-25% lower than AFD followed by CHAPS/urea protein extraction [4]. This recovery yield following direct sample homogenization in TRIzol^®^ was higher than the yield we report, although the approaches are not entirely comparable. Protein extraction from rat spinal cord samples used in that study was performed from pooled organic and aqueous fractions, following RNA extraction and precipitation, whereas in our study proteins were only extracted from the organic phases of TRIzol^®^-Chloform processed samples, following removal of the aqueous phase. This may account for the differences in protein yield. Importantly, in the same study, and despite marked differences in protein recovery yield of sample homogenates processed directly in TRIzol^®^ or by AFD followed by TRIzol^®^, these samples were evaluated by microarray and 2DE analyses. Furthermore, it was shown that the two homogenization strategies were effectively interchangeable from the perspective of the genomics analysis, and that TRIzol^®^ provided effective protein recovery, with only minor alterations to the resolved proteome. In fact, at the level of 2DE, both sample processing methods resulted in high quality and resolution proteomic maps of the biological material with only 58 proteins found significantly different between both preparations, where 14 proteins were only detected by using a single sample preparation, and the remaining 44 proteins were detected in both preparations but differed significantly in relative abundance, which may also be attributable to sample to sample differences in protein abundance [4].

The protein patterns we obtained from total protein extract separation in 8% SDS polyacrylamide gel, following Ponceau or Coomassie staining, clearly demonstrated that the modified TRIzol protocol allows a staining pattern for recovered proteins similar to that obtained following mirVana™ PARIS™ and standardized laboratory protein extraction protocols (Figure 2, lanes 5 and 6, versus lanes 1 to 4). Further, the protein patterns and recovery yield obtained from the modified TRIzol protocol without sonication, and from the TRIzol^®^ and TRIzol^®^LS manufacturer’s protocol were not representative of the protein content in the samples. In fact, they displayed different staining patterns from those obtained with the modified TRIzol protocol, and with the mirVana™ PARIS™ and standardized laboratory protocols (Figures 2 and 3, lanes 7–10 and 11–14, respectively, versus lanes 1–6). However, 1D gels may provide a superficial indication of protein recovery and cannot attest as to whether or not selective extraction of particular individual proteins may occur. Nevertheless, using a similar input of a particular tissue type, putative selective expression of a given protein would be more likely to derive from extraction method specificities rather than derive from sample to sample abundance. Therefore, the applicability of this method to the evaluation of steady-state expression of the whole proteome, particularly for undetectable proteins or to quantitative analysis, needs further testing.

**Figure 4:**
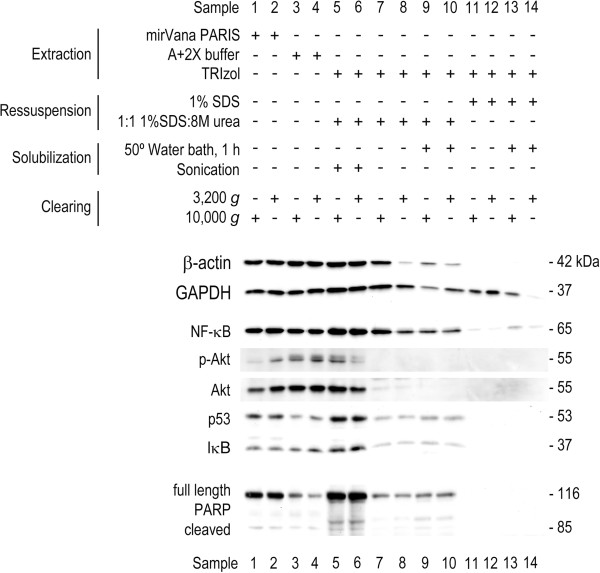
**Performance of the modified TRIzol protocol evaluated by immunoblotting.** To compare the modified TRIzol protocol versus the TRIzol^®^ manufacturer’s, mirVana™ PARIS™ kit and standardized laboratory (A+2X Buffers) protocols, 10^6^ HCT116 cells were plated in 14 independent 100 mm plates (one per experimental setting: lanes 1 to 14), and cells were processed for total protein extraction 48 h after plating. Next, 40 μg of total protein extracts were separated on 8% SDS-PAGE and transferred onto nitrocellulose membrane. Immunoblot analysis of steady-state expression levels of housekeeping proteins (β-actin and GAPDH), NF-κB, IκB, p-Akt, Akt, p53, and nuclear PARP.

Importantly, to validate the performance of the modified TRIzol protocol, we evaluated the steady-state expression levels of the housekeeping proteins GAPDH and β-actin. In addition, we also evaluated the steady-state expression levels of the nuclear protein PARP, and of other proteins whose expression changes have been previously reported in cancer, including NF-κB, IκB, p53 and Akt. Our results demonstrated that the steady-state expression levels of β-actin and GAPDH obtained with the modified TRIzol protocol are very similar to those obtained with mirVana™ PARIS™ and standardized laboratory protocols (Figure 4, lanes 5 and 6, versus lanes 1–4). However, when total proteins are obtained by the modified TRIzol protocol without sonication, and by the TRIzol^®^ manufacturer’s protocol, we found striking differences (Figure 4, lanes 7–14 versus lanes 1–6). Worthy of note, the abundant cellular protein β-actin was undetectable in protein samples obtained with the TRIzol^®^ manufacturer’s protocol, and almost undetectable in protein samples that were not sonicated (Figure 4, lanes 11–14, and lanes 7–10, respectively), thus highlighting the poor, and unreliable, protein recovery performance of these methods, in comparison with the modified TRIzol protocol.

It is known that multiple proteins involved in the regulation of key cellular functions are deregulated in many diseases, including cancer. To further test the performance of the modified protein extraction protocol, we evaluated the steady-state levels of NF-κB, IκB, p53, p-Akt and Akt. Our results clearly showed that we were able to detect these proteins, with comparable sensitivity to that obtained using mirVana™ PARIS™ and standardized laboratory protocols (Figure 4, lanes 5 and 6 versus lanes 1–4); and improved sensitivity compared to the TRIzol^®^ manufacturer`s protocol and to the modified TRIzol protocol without sonication (Figure 4, lanes 5 and 6 versus lanes 11–14 and 7–10, respectively). Furthermore, it is also possible to detect phosphorylated proteins, such as p-Akt, illustrating the ability to detect these post-translational protein modifications in TRIzol^®^ treated samples using the modified TRIzol protocol. Finally, the modified TRIzol protocol is suitable to detect nuclear PARP (Figure 4, lanes 5 and 6). PARP is undetected from total protein extracts processed by the TRIzol^®^ manufacturer’s protocol, and is hardly detectable by using modified TRIzol protocol without sonication (Figure 4, lanes 11–14 and 7–10, respectively). Worthy of note, PARP is a 116 kDa nuclear protein, cleaved by caspase-3 upon apoptotic stimuli, yielding an 85 kDa fragment. With the modified TRIzol protocol, it was possible to detect the full length and the cleaved fragment, with increased sensitivity compared to other protocols.

### Ponceau S staining as immunobloting loading control

**Figure 5:**
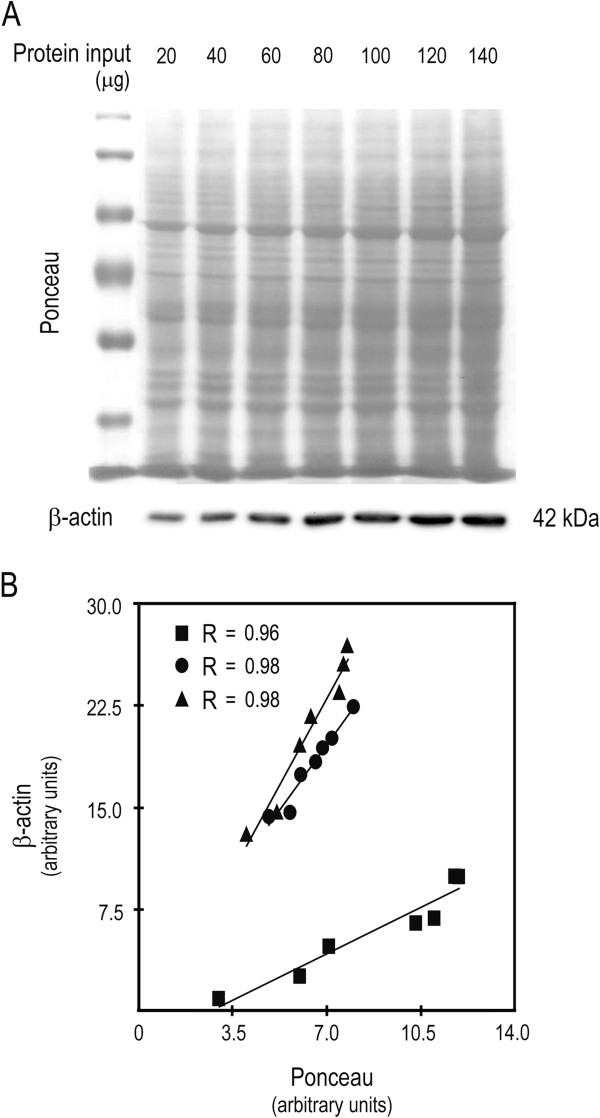
**Ponceau S staining is comparable to β-actin as a loading control for immunobloting.** HCT116 cells were plated at 10^6^ per 100 mm plate, and cells were processed for protein extraction with standardized laboratory A+2X protocol, 48 h after plating. Increasing input of protein extract, from 20 to 140 μg with 20 μg increments, were next separated in 8% SDS-PAGE followed by transfer to nitrocellulose membrane, Ponceau S staining and β-actin steady-state expression evaluation. (**A**) Representative Ponceau S staining and steady-state expression levels of β-actin. (**B**) Correlation between Ponceau S and β-actin relative quantification, from three independent experiments.

We and others have used Ponceau S staining as a loading control for western blot [14-16]. This is particularly relevant when experiments require protein separation in low percentage SDS polyacrylamide gels, in which the typical housekeeping controls migrate out of the gel. In addition, it is also important in conditions in which the typical housekeeping proteins are not adequate controls, due to changes in their steady-state expression levels, including during stem cell differentiation [14,16]. In this study, we validated the performance and used Ponceau staining as a normalization control for immunoblot analysis. For this purpose, we separated total protein extracts in 8% SDS polyacrylamide gel, using 20 to 140 μg of total protein extracts as input, and evaluated the steady-state expression of the housekeeping loading control, β-actin (Figure 5A). We next quantitated Ponceau and β-actin signals, and showed a strong correlation between both signals, for the range of protein inputs tested (20 to 140 μg, with 20 μg increments; R = 0.97 ± 0.007) (Figure 5B). We also evaluated the ratio of β-actin over Ponceau (β-actin/ponceau), for several immunoblots with 5–10 samples each, and performed linear regression analysis. The equation slopes provided a measure of variability between β-actin and Ponceau staining as normalization controls. The closer the slope is to zero, the higher is the concordance between both variables. Our results provided a mean slope of 0.0003 ± 0.0003 (data not shown), highlighting that sample normalization with Ponceau staining is equally effective as the regular sample normalization with the housekeeping β-actin.

### Steady-state protein expression levels in human colon cancer

**Figure 6:**
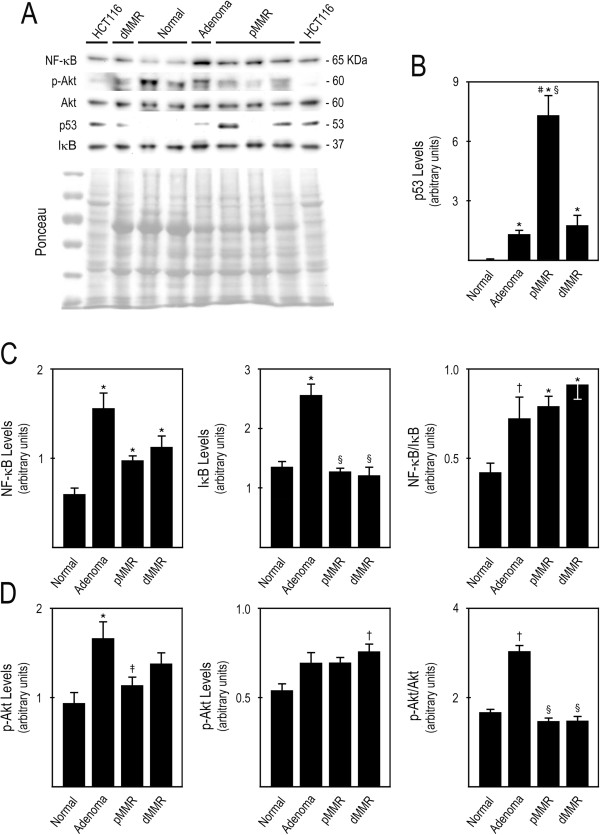
**Steady-state protein expression levels in human colon cancer samples.** Protein extracts were obtained from human colon cancer samples stored in TRIzol^®^-chloroform samples at −80°C for 2 years. Forty μg of total protein extracts were separated in 8% SDS-PAGE, transferred to nitrocellulose membrane and evaluated for the steady-state protein expression levels. (**A**) Representative imunoblots of steady-state expression levels of p53, NF-κB, IκB, p-Akt and Akt, in HCT116 colon carcinoma cells, normal colon, colon adenomas and colon pMMR and dMMR carcinomas. (**B**) p53 protein steady-state levels in normal colon, colon adenomas, and pMMR and dMMR colon carcinomas; (**C**) NF-κB, IκB, and NF-κB/IκB ratio; and (**D**) p-Akt, Akt, and p-Akt/Akt ratio. Results are expressed as mean ± SEM for similar samples. *p < 0.01 and †p < 0.05 from normal colon; §p < 0.01 and ‡p < 0.05 from colon adenomas; #p < 0.01 from colon sporadic dMMR carcinomas.

In this study, using the modified TRIzol protocol and Ponceau as normalization control, we evaluated the steady-state protein expression of p53, NF-κB and its inhibitor IκB, and p-Akt and Akt, in a large set of well-defined human colon samples, including normal colonic mucosa, tubolovillous adenomas, pMMR and sporadic dMMR adenocarcinomas. These samples were previously processed with Trizol^®^LS for RNA extraction, which was used for the evaluation of miRNA expression profiles [7]. The TRIzol^®^-chloroform fractions of these human colon samples were stored at −80°C for 2 years, before they were processed for total protein extraction following our modified TRIzol protocol. We next used these protein extracts to evaluate if following 8% SDS polyacrylamide gel separation, immunoblot detection of these proteins reproduced their reported steady-state expression levels in CC. Following the modified TRIzol protocol, these proteins were easily detectable (Figure 6A), allowing the evaluation of the steady-state levels of p53, NF-κB and its inhibitor IκB, and p-Akt and Akt.

Loss of p53 function by mutation is a common event in cancer, at rates varying between 10% and close to 100%. These mutational events have been reported to occur at different phases of the multistep process of malignant transformation [17]. However, the loss of p53 function in CC is usually associated with adenoma to carcinoma transition [18,19]. In most cases, the p53 gene mutation gives rise to a stable mutant protein, which tends to accumulate in the cell [17]. Loss of heterozygosity is believed to be the main inactivation event on one of the alleles of tumor suppressor genes in cancer, where approximately 70% of CC displays 17p LOH. In most cases of CC, the main target of 17p LOH is the p53 gene; and ~ 85% of the somatic mutations carried in the remaining p53 allele are missense mutations [20]. In addition, the loss of functional p53 in adenomas and dMMR carcinomas represents the minority of cases, in contrast to pMMR carcinomas, where p53 is frequently mutated [19,21]. The lower rate of p53 mutation might also be related with the better prognosis associated with CC presenting MSI (dMMR) in contrast to CC exhibiting an intact mismatch repair system (pMMR) [22]. In addition, p53 is critically involved in drug-induced apoptosis, in which it is up-regulated and translocated to mitochondria following HCT116 colon cancer cells exposure to 5-fluorouracil [23].

Our results showed increased p53 steady-state levels in adenomas as well as in pMMR and dMMR tumors (p < 0.01) (Figure 6B). Importantly, p53 levels in adenomas and dMMR carcinomas were much lower than those found in pMMR carcinomas, which might be associated with an attempt to control cell growth, rather than reflect p53 mutations. The high steady-state levels of p53 found in pMMR carcinomas were possibly related to the accumulation of stable mutant forms of p53. The present results are in accordance with the literature, and suggest that the loss of functional p53 is indeed involved in the adenoma to carcinoma transition, and that the status of the mismatch repair system is strongly correlated with the frequency of p53 mutations [24].

The NF-κB and PI3K/Akt signaling pathways are frequently deregulated in several types of cancer, including CC [13]. Abnormal NF-κB and Akt activation and overexpression was shown to play an important role in cellular transformation, and may lead to uncontrolled cell growth and survival, apoptosis suppression, and increased angiogenesis and metastasis [25-27]. The overexpression and activation of NF-κB in CC have been particularly associated with colon tumor progression [28,29]. In turn, Akt overactivation is typically more common then its overexpression, due mainly to the overexpression of EGFR in this type of cancer [27]. In addition, the involvement of Akt in sporadic colon carcinogenesis has already been reported as an important early event [30]. For the evaluation of the NF-κB and Akt signaling pathways we analysed NF-κB and IκB, and p-Akt and Akt steady-state levels, respectively. To assess NF-κB and Akt activation we calculated the ratio of NF-κB over IκB, and p-Akt over Akt, respectively, in normal colonic mucosa, colon adenomas, and colon pMMR and dMMR carcinomas. The results clearly show that NF-κB is overexpressed in colon adenomas, pMMR carcinomas, and also dMMR carcinomas, compared to normal colon tissue (p < 0.01) (Figure 6C, left panel). Further, our data also indicated that NF-κB is aberrantly overactivated in colon adenomas (p < 0.05) and in pMMR and dMMR tumors (p < 0.01), compared to normal colonic tissue (Figure 6C, right panel). In turn, our results showed that p-Akt steady-state levels were significantly increased in adenomas (p < 0.01) and displayed a trend for increase in both pMMR and dMMR carcinomas compared to normal colon tissue (Figure 6D, left panel). Akt levels displayed a trend for increase in adenomas and pMMR carcinomas, and were found significantly elevated in dMMR carcinomas (p < 0.01), compared to normal colon tissue (Figure 6C, middle panel). Importantly, Akt activation was significantly increased in adenomas compared to normal tissue and to pMMR and dMMR carcinomas (p< 0.01) (Figure 6D, right panel). Therefore, our results indicated aberrant NF-κB signaling in colon adenomas and carcinomas, and suggested that overactivation of Akt signaling may represent an early event in CC. Collectively, these studies showed that our modified TRIzol protein extraction protocol is an adequate technique to evaluate the steady-state levels of proteins in human cancer, representing a fast, cost-effective alternative to allow adequate protein extraction from samples that were simultaneously processed for nucleic acid extraction and following prolonged storage, which significantly expands the current known applicability of TRIzol^®^ protein extracts. Further, our report significantly highlights the relevance, and ease of access, of potentially relevant information obtainable from protein expression analyses by immunoblot, following protein extraction from organic fractions of routine TRIzol^®^ processed samples, which are usually discarded. In studies in which tissue samples have already been collected and TRIzol^®^- or TRIzol^®^LS-processed, our method provides a simple, fast and cost-effective way of accessing relative protein expression by immunoblot.

### Steady-state protein expression levels in mouse brain

**Figure 7:**
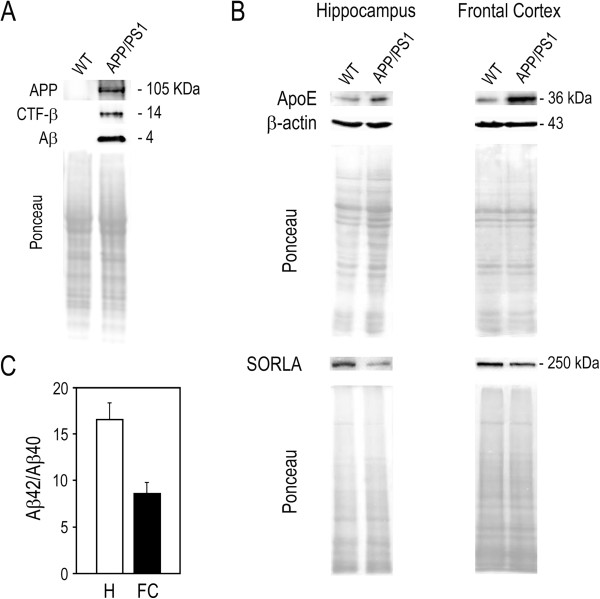
**Steady-state protein expression levels in mouse brain samples.** Protein extracts were obtained from APP/PS1 and wild-type mice brain samples stored in TRIzol^®^-chloroform samples at −80°C for 3 months. (**A**) Sixty μg of total protein extracts from wild-type (WT) and APP/PS1 mice hippocampus were electrophoretically separated in 10-20% Tris-Tricine gels, transferred to nitrocellulose membrane and evaluated for the steady-state protein expression levels of human APP, APP-C-terminal fragment β (CTF-β) and amyloid-β (Aβ) peptide. Ponceau S staining was used as loading control. (**B**) Sixty μg of total protein extracts from WT and APP/PS1 mice hippocampus and frontal cortex were separated in 6 and 12% SDS-PAGE, transferred to nitrocellulose membrane and evaluated for the steady-state protein expression levels of apoE and SORLA. β-Actin and/or Ponceau S staining were used as loading controls. (**C**) Ten ng of total protein extracts from APP/PS1 mice hippocampus and frontal cortex (H and FC, respectively) were used to measure human total Aβ_1–40_ or Aβ_1–42_ content by sandwich ELISA, and to determine individual Aβ_1–42_ to Aβ_1–40_ ratio. Data are mean ± SEM from 5 mice in each brain region.

In AD, Aβ peptides are generated by successive proteolysis of APP that is initially cleaved by β-secretase, and subsequently by γ-secretase [31-33]. In this study, we evaluated APP and APP-C-terminal fragment cleaved by β-secretase (CTF-β) steady-state protein levels, using proteins collected from brain tissues of APP/PS1 mice and wild-type littermates, with the modified TRIzol protocol and Ponceau as normalization control. For protein isolation, we used the TRIzol^®^-chloroform extracts stored at −80°C for 3 months following RNA extraction [9]. We next performed electrophoresis in 10-20% Tris-Tricine gels, followed by immunodetection of human APP full-length, APP-CTF-β, and Aβ. As expected, APP and its fragments were only detected in APP/PS1 mice (Figure 7A).

The major genetic risk factor for late-onset AD is the presence of the ε4 allele of the apolipoprotein E (ApoE) gene [34], which encodes a protein that contributes to AD pathogenesis by modulating the metabolism and aggregation of Aβ peptide and by directly regulating brain lipid metabolism and synaptic function through apoE receptors (reviewed in [35]). Indeed, ApoE itself binds Aβ with high specificity and is present in AD senile plaques [36]. In contrast, sortilin-related receptor with A-type repeats (SORLA), an ApoE-binding protein, may play a protective role in AD by reducing APP processing and generation of Aβ peptides [37]. Accordingly, loss of SORLA occurs in AD brain patients [38]. Therefore, given the role of lipid mediators in modulating Aβ metabolism [39], we assessed ApoE and SORLA protein steady-state levels. Western blot analysis of total protein extracts demonstrated increased steady-state expression of ApoE in the hippocampus and frontal cortex of APP/PS1 mice compared to control wild-type mice, whereas decreased expression of SORLA was observed in both cerebral regions of APP/PS1 mice (Figure 7B). Finally, we analyzed human Aβ_1-42_ and Aβ_1-40_ levels in the hippocampus and frontal cortex of APP/PS1 mice brains by sandwich ELISA (Figure 7C). Aβ_1-42_ to Aβ_1-40_ ratio is an important factor in determining the fibrillogenesis, toxicity, and pathological distribution of Aβ, and appears to correlate with AD pathology [40]. In APP/PS1 mice hemibrains, Aβ_1-42_ to Aβ_1-40_ ratio was shown to increase with AD progression from 1.6 to 5 in 1- and 8-month-old mice, respectively [10]. Our results on dissected hippocampus and frontal cortex are in agreement with the high value expected for Aβ_1-42_ to Aβ_1-40_ ratio. Therefore, the modified TRIzol protocol for protein extraction provides an adequate tool for protein analysis in brain tissue samples, coupled to RNA analysis. This was particularly apparent for the specific detection of intracellular Aβ accumulation and APP processing, the detection of variations in key proteins associated with AD pathogenesis between wild-type and transgenic mice samples, and the measurement of Aβ_1-42_ and Aβ_1-40_ levels by ELISA. Similar sensitivity of the modified TRIzol protocol was obtained in experiments using primary mouse cortical neurons incubated with Aβ (data not shown).

## Conclusions

Our results clearly demonstrated that simple modifications introduced to the TRIzol^®^ manufacturer’s protocol, consisting of protein resuspension using 1:1 solution of 1% SDS and 8 M urea followed by sonication, greatly improved the protein recovery performance. This modified protocol allows higher protein recovery yield, while producing protein patterns comparable to those obtained by other extraction methods, following SDS-PAGE and Coomassie or Ponceau staining. In addition, we demonstrated the importance of the sample sonication step for efficient ressuspension of the recovered protein pellet. Our modified TRIzol protocol also allowed the detection of post-translational modifications, namely protein phosphorylation, and cellular protein distribution. In addition, our results further validated the use of Ponceau as a loading control, demonstrating that Ponceau S staining and β-actin relative quantification provided comparable performance, which is particularly relevant when regular housekeeping proteins are inadequate controls, e.g., during stem cell differentiation. Furthermore, our modified method allowed the detection and relative quantification of steady-state expression levels of important proteins deregulated in disease conditions, reproducing the current literature. However, we cannot conclude if our method would be suitable for quantitative protein analysis, nor for mass spectrometry since high concentrations of urea may lead to protein carbamylation. Collectively, we present a simple, fast, and inexpensive modified TRIzol protocol for protein extraction, which allows the recovery of total protein extracts from multiple sources, and its applicability to good quality immunoblot detection of the steady state protein expression levels from samples that have simultaneously been processed for nucleic acid extraction, following prolonged storage in TRIzol^®^-chloroform at −80°.

## Competing interests

The authors declare that they have no competing interests.

## Authors’ contribution

CMPR, PMB conceived, designed and coordinated the experiments. AESS, JDA, AFN, SEG, DMP, PMR, PMB performed the experiments. AESS, JDA, AFN, ACL, RD, CJS, SNT, CMPR, PMB analyzed the data. AESS, CMPR, PMB wrote the manuscript. All authors read and approved the final manuscript.

## Finantial support

This study was supported by Fundação para a Ciência e a Tecnologia (FCT), through grants PTDC/SAU-GMG/099162/2008, and PTDC/SAU-NMC/117877/2010 (to C.M.P.R.) and PEst-OE/SAU/UI4013/2011, and by Sociedade Portuguesa de Gastrenterologia. A.E.S.S., A.F.N. and J.D.A. were recipients of PhD fellowship SFRH/BD/79356/2011, and Postdoctoral fellowships SFRH/BPD/34603/2007 and SFRH/BPD/47376/2008, respectively, from FCT. The funders had no role in study design, data collection and analysis, decision to publish, or preparation of the manuscript.
